# ARHI is a novel epigenetic silenced tumor suppressor in sporadic pheochromocytoma

**DOI:** 10.18632/oncotarget.21149

**Published:** 2017-09-21

**Authors:** Dong Wang, Li Song, Liang Wang, Lianmei Zhao, Bai Xiang, Ying Li, Baoen Shan, Jing Liu

**Affiliations:** ^1^ Department of Urology, Peking Union Medical College Hospital, Beijing 100730, P.R. China; ^2^ Department of Biochemistry and Molecular Biology, College of Basic Medicine, Hebei Medical University, Shijiazhuang 050017, P.R. China; ^3^ State-Level International Cooperation Base, Regenerative Medicine Center, The First Affiliated Hospital of Dalian Medical University, Dalian 116011, P.R. China; ^4^ Research Center, The Fourth Hospital of Hebei Medical University, Shijiazhuang 050011, P.R. China; ^5^ Department of Pharmaceutics, School of Pharmaceutical Sciences, Hebei Medical University, Shijiazhuang 050017, P.R. China

**Keywords:** ARHI, epigenetic tumor suppressor, sporadic pheochromocytoma, copy number deletion, p27Kip1

## Abstract

Pheochromocytoma (PCC) is related to germline mutations in 12 susceptibility genes. Although comparative genomic hybridization array has revealed some putative tumor suppressor genes on the short arm of chromosome 1 that are likely to be involved in PCC tumorigenesis, the molecules involved, except for those encoded by known susceptibility genes, have not been found in the generation of sporadic tumors. In the present work, we first identified that the unmethylated allele of Aplasia Ras homolog member I (*ARHI*) was deleted in most PCC tumors which retained a hypermethylated copy, while its mRNA level was significantly correlated with the unmethylated copy. De-methylation experiments confirmed that expression of ARHI was also regulated by the methylation level of the remaining allele. Furthermore, ARHI overexpression inhibited cell proliferation, with cell cycle arrest and induction of apoptosis, in ARHI-negative primary human PCC cells, whereas knockdown of ARHI demonstrated the opposite effect in ARHI-positive primary human PCC cells. Finally, we demonstrated that ARHI has the ability to suppress pAKT and pErK1/2, to promote the expression of p21^Waf1/Cip1^ and p27^Kip1^, and also to increase p27^Kip1^ protein stability. In summary, ARHI was silenced or downregulated in PCC tissues harboring only one hypermethylated allele. ARHI contributes to tumor suppression through inhibition of PI3K/AKT and MAKP/ERK pathways, to upregulate cell cycle inhibitors such as p27^Kip1^. We therefore reasoned that ARHI is a novel epigenetic silenced tumor suppressor gene on chromosome 1p that is involved in sporadic PCC tumorigenesis.

## INTRODUCTION

Pheochromocytomas (PCC) are rare, usually benign endocrine tumors arising from chromaffin cells that produce catecholamines in the adrenal medulla. About one-third of PCC occur in a hereditary context and are characterized by germline mutations in key susceptibility genes including the RET and HIF2A proto-oncogene and the VHL, NF1, SDHx (SDHA, SDHB, SDHC, SDHD, SDHAF2), FH, TMEM127 and MAX tumor suppressor genes (TSGs) [1-11]. Somatic mutations in HIF2A and NF1 have been discovered recently in a further 30% of PCC [12-14]. To our knowledge, major germline and somatic mutations can be ruled out in about 40% of PCC, and the additional events involved in tumorigenesis without known abnormalities in the genomic background are still unclear.

PCC seem to be more commonly associated with genomic variation than any other cancer type [15, 16]. DNA copy number deletion is “one hit”, and induces TSGs (tumor suppressor genes) silencing in PCC. Genome-wide screening for PCC susceptibility loci has identified some minimal overlapping regions (MORs) on chromosome 1p where one or more novel TSGs involved in PCC are located [17-19]. However, to date no specific target genes have been identified with functional significance, and the genetic mechanisms underlying the tumorigenesis of sporadic PCC remain relatively poorly characterized.

Aplasia Ras homolog member I (ARHI; also known as DIRAS3) is an imprinted tumor suppressor gene which encodes a 26 kDa GTPase with homology to Ras. The ARHI mRNA level has been found to be downregulated significantly in ovarian cancer and breast cancer [20-22]. ARHI overexpression at the physiologic level induces inhibition of cell proliferation, acceleration of apoptosis and increased autophagy [23]. ARHI is located on chromosome 1p31, which is a very common deletion locus in sporadic PCC [19, 24-26]. Moreover, as an imprinted gene, Hypermethylation of the ARHI allele can be achieved with “one hit” during tumorigenesis [27-29]. Recent data have also demonstrated that ARHI inhibits the PI3K/AKT and Ras/MAPK signal transduction pathways, which also are two major dysfunctional pathways in most sporadic PCC [30, 31]. Therefore, we suspect that ARHI satisfies the “two hits” hypothesis and is a novel tumor suppressor in sporadic PCC.

In this study, we found the functional copy number of ARHI to be frequently deleted in most PCC tumors; the promoter of the other allele was nearly 100% methylated and its expression decreased in samples of sporadic PCC. Gain- and loss-of-function studies demonstrated that ARHI inhibits proliferation of primary human pheochromocytoma cells (PHPC), with cell cycle arrest, promoting p27^Kip1^ expression and stability through reduction of pAKT and pErK1/2. In conclusion, ARHI has been identified as a novel epigenetic tumor suppressor gene located on chromosome 1p, and our results show that it has an important function in sporadic PCC tumorigenesis.

## RESULTS

### ARHI copy number deletion and underexpression occur frequently in sporadic PCC

**Figure 1:**
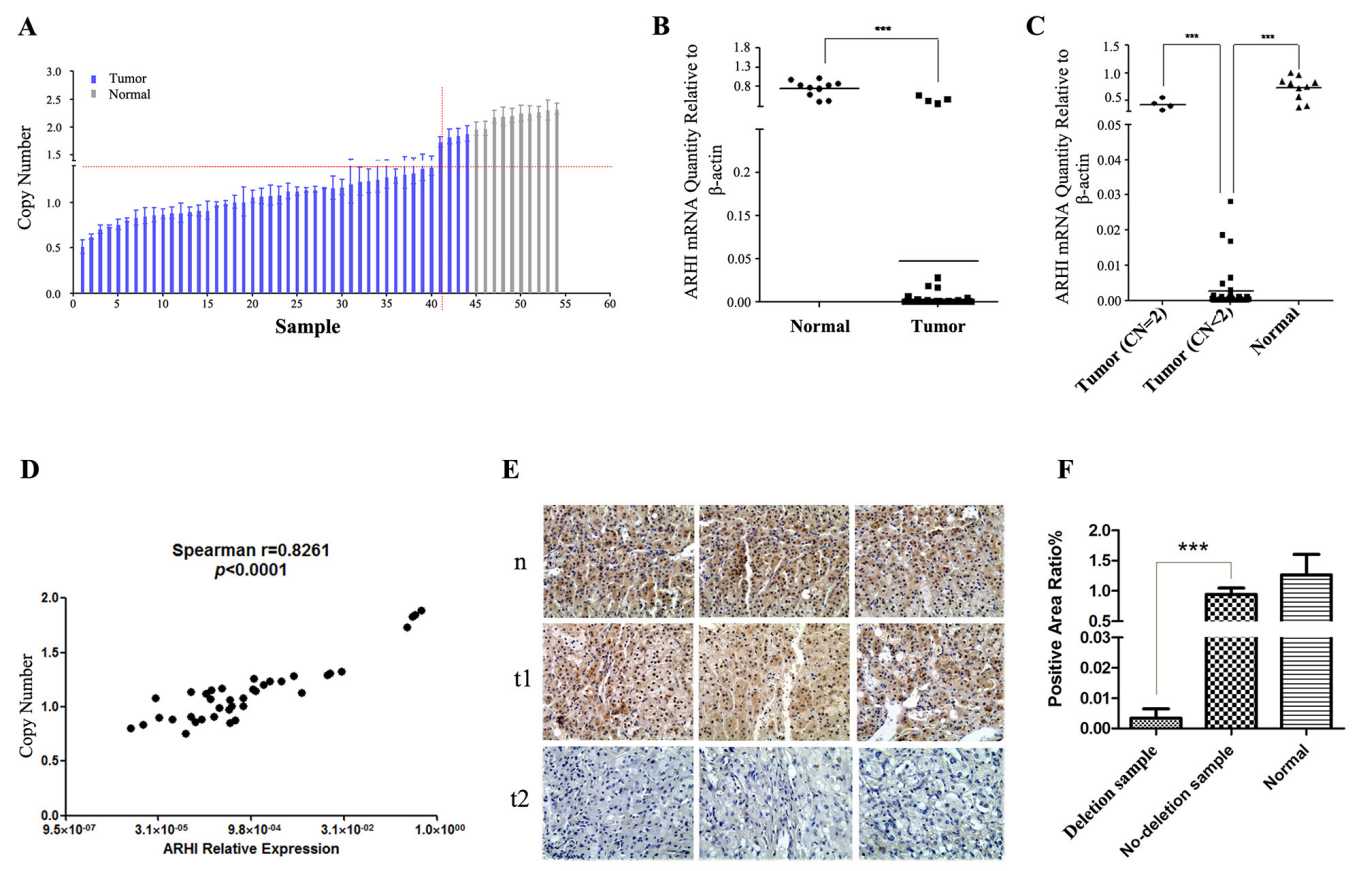
ARHI lost a functional allele and downregulated in sporadic PCC **(A)** ARHI copy number variation in PCC tumors. qRT-PCR was carried out with 10 ng per reaction of the same genomic DNA used in the promoter analysis. All samples were then normalized to C2 and the copy number in blood samples was set to 2. Copy number relative ratio <1.4 was determined as copy number deletion, and copy number relative ratio >2.6 was recorded as amplification. **(B)** Expression of ARHI mRNA in normal adjacent tissue (n=10) and primary PCC (n=38). When compared with normal tissue, ARHI is significantly reduced in the PCC (***p<0.0001, two-sided Mann Whitney test). **(C)** ARHI mRNA expression levels were significantly lower in copy number deletion tumors (n=38) when compared with those that carried normal copy number (n=10)(***p=0.0013, two-sided Mann Whitney test). **(D)** Correlation between *ARHI* copy number and its expression in PCC samples (n=38). **(E)** Representative results for three PCC specimens with ARHI deletion (bottom described as t2), three PCC without deletion of ARHI (middle described as t1) and corresponding noncancerous adrenal medulla (top described as n) by immunohistochemical staining with anti-ARHI antibody. ARHI was stained brown with granules and located in the cytoplasm and nuclei. **(F)** Schematic analysis of positive staining area of IHC in Figure 1E, the error bars are represented as mean ± SD.

We expanded our analysis to a panel of 44 PCC tumors. Of these tumors, 3 tumors contained HIF2A somatic mutations and 7 tumors with RET somatic mutations, others are truly sporadic cases that contain no mutations in known susceptibility genes (Supplementary Table 1). 40 (90%) tumors had a copy number deletion of the ARHI genomic locus (Figure 1A). Furthermore, examination of matched gene expression revealed a significant reduction in ARHI mRNA levels in tumors, and this was exaggerated in samples with copy number deletion (Figure 1B and 1C). Simultaneously, we also demonstrated a strong correlation between ARHI mRNA expression and its copy number variation (Figure 1D). The degree of immunopositivity in clinical samples was assessed as the percentage of area positive for ARHI. Positive staining was predominantly observed in normal cells (200×) (Figure 1E). PCC tumors with ARHI deletion showed significantly less staining than those without ARHI deletion and normal tissue (Figure 1E and 1F). This result indicates that ARHI protein is significantly decreased in ARHI-deleted PCC tumor cells. Microarray data GSE38525 [32] and sequencing data E-MTAB-591 [10] also confirmed ARHI deletion and downregulation in sporadic PCC (Supplementary Figures 1 and 2). These results show that ARHI is a target of the 1p31.3 deletion, and that ARHI downregulation is a very common event in sporadic PCC. In addition, inactivation of ARHI may confer a survival advantage on adrenal medullar cells during PCC tumorigenesis.

### The promoter hypermethylation of ARHI silences its expression in PCC

**Figure 2:**
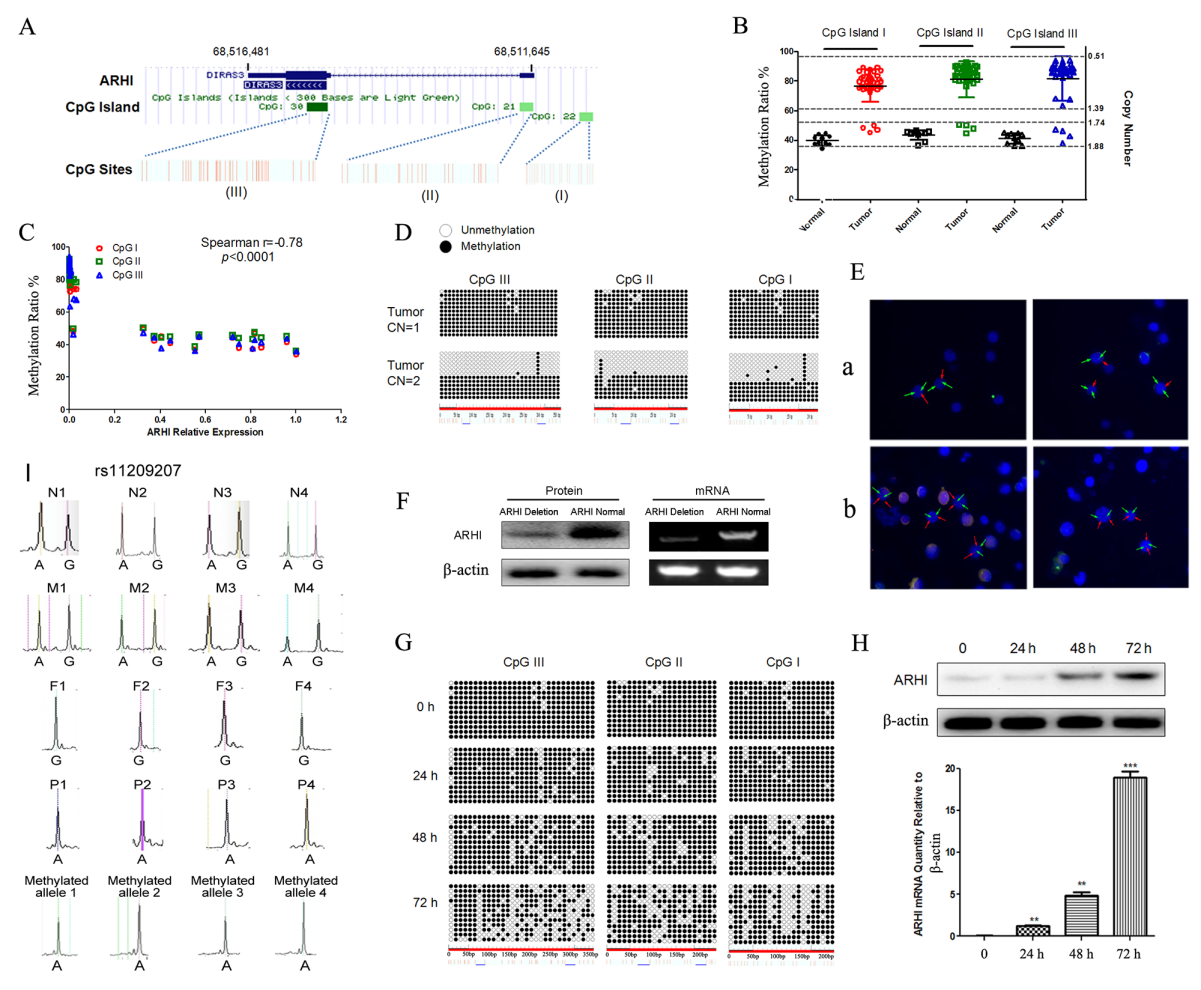
ARHI retained a hypermethylation copy in sporadic PCC **(A)** Schematic representations for the localization of CpG islands. Bottom boxes from left to right indicate CpG islands I, II, and III, respectively, and the red bar in shows the CpG sites. The corresponding numbers indicated the location in the genome from the UCSC database. **(B)** The ARHI promoter included three CpG islands; the methylation ratios were detected by EpiTYPER methylation analysis. The methylation ratios were significantly higher in PCC tumors than normal adrenal tissues. The methylation ratios of the three CpG islands were obviously higher in the tumors with deleted copy numbers of ARHI when compared with those that carried normal copy numbers of ARHI. **(C)** Correlation between *ARHI* promoter CpG islands and their expression in PCC samples (n=38). **(D)** Summary of bisulfite-treated genomic DNA sequencing of PCC samples dependent on ARHI deletion, where the amplified region includes all three CpG islands; 73 CpG dinucleotides (CpGs), represented by circles located on the region, were analyzed by DNA sequencing. Black and white circles represent the methylated and unmethylated CpG dinucleotides, respectively. Each line represents the DNA sequence of a random clone, of which black and white circles represent unmethylated and methylated CpG sites of these regions, respectively. **(E)** Fluorescence *in situ* hybridization studies in Subjects 1 and 2. In Subjects 1, the 1chr.p31.3 (ARHI) labeled with Rhodamine BAC clone showed 2 copies (panel a) while the “control”chr.1q21 labeled with FITC BAC clone showed normal hybridization pattern in nuclei (Green). Subjects 2 showed the ARHI deletion (panel b) detected by the Rhodamine BAC clone (arrow). The FITC BAC clone was the control probe. **(F)** PHPC with ARHI copy number deletion (without endogenous ARHI expression) and with normal ARHI copy number were used to detect the methylation status using bisulfite-treated genomic DNA sequencing. Western blot and RT-PCR were used to determine whether ARHI was expressed at protein and mRNA levels. **(G)** Effect of DAC expression on methylation status of the ARHI gene promoter. DNA from control or DAC-treated PHPC with negative ARHI expression were collected at the indicated time points, cloned and sequenced to detect CpG-island methylation of the ARHI promoter. **(H)** ARHI-negative PHPC were treated with DAC, after 24h, 48h, 72h; ARHI was detected using RT-PCR and western blot, the error bars are represented as mean ± SD. **(I)** ARHI methylated allele analysis and its maternal imprinting in PCC tumors. SNP rs11209207 of normal DNA from 4 PCC patients (lane 1:N1-N4): one allele is lost in tumor DNA (lane 4:P1-P4); the retained allele is methylated (lane 5:Methylated allele1-4). Genotype of SNP rs11209207 of 4 families was shown. Maternal (lane 2:M1-M4); paternal (lane 3:F1-F4).

ARHI is an imprinted TSG involved in various types of cancer, and its normal expression occurs from the paternal allele. In most sporadic PCC, one copy of ARHI is deleted and its expression almost absent. Therefore, we suspect that the inactivated allele of ARHI is retained, of which the promoter is hypermethylated. To test our hypothesis, we initially used the EpiTYPER MassARRAY System (Sequenom, USA) for quantitative DNA methylation analysis of ARHI promoter CpG islands. We analyzed all three CpG islands of ARHI separately and found aberrant hypermethylation of ARHI in sporadic PCC, when compared with normal tissue (Figure 2A and 2B). In this cohort, 4 samples with ARHI normal copy number nearly (3 samples) contained HIF2A mutations. There is a negative correlation between ARHI promoter CpG island hypermethylation and its expression in PCCs (Figure 2C). Furthermore, ARHI copy number in PHPC was confirmed by Fluorescence in situ hybridization and we found that endogenous ARHI was not detected in PHCP with only a hypermethylated allele and it showed almost 100% methylation. ARHI was expressed at a high level in PHCP with two alleles, including one unmethylated (Figure 2D, 2E, and 2F). To determine whether ARHI methylation silences its mRNA expression, PHPC from fresh human PCC was treated with the DNMT1 inhibitor 5-aza-2′-deoxycytidine (DAC). After DAC treatment, PHPC demonstrated a gradual demethylation and a significant increase in mRNA and protein (Figure 2G and 2H). ARHI expression depletion in PCC tumors could result from loss of heterozygosity (LOH) of the non-imprinted allele (Supplementary Figure 1). To test this possibility, we chose 4 PCC patients and their mother is A/G herozygous in SNP rs11209207, their father is G homozygous. Then both LOH and imprinting could be evaluated in the 4 available PCC families. We compared the SNP rs11209207 in normal and tumor DNA from the same patient, only one retained allele which was methylated can be amplified after genomic DNA digested by the methylation-sensitive restriction enzyme *BstU*I (Takara) in all four patients (Figure 2I). The results showed that ARHI may also be an important epigenetic tumor related gene in PCC tumorigenesis.

### ARHI decreases PHPC proliferation

**Figure 3:**
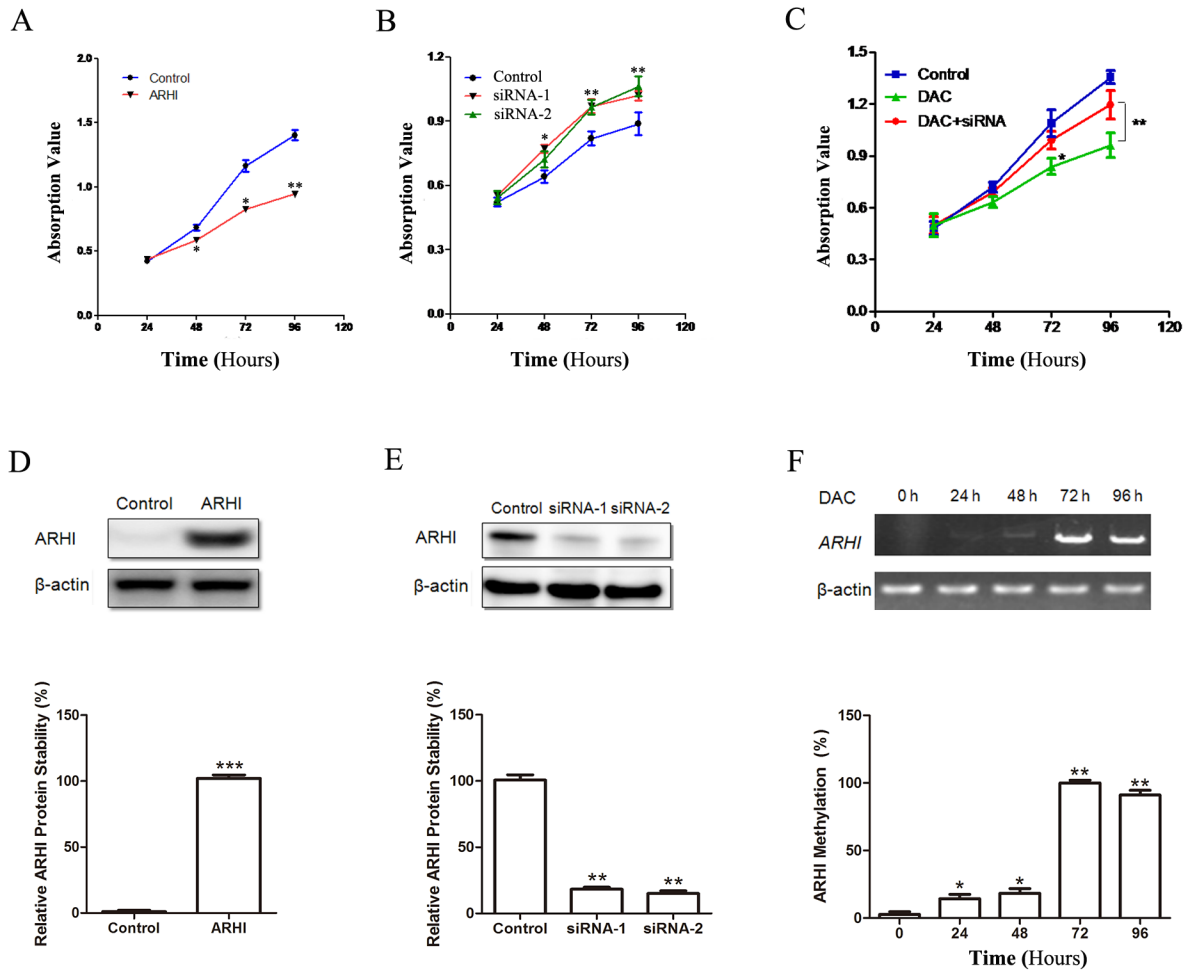
ARHI inhibited PHCP proliferation **(A)** PHPC with negative endogenous ARHI were transfected with pcDNA3.1-ARHI; cell proliferation was detected at different time points. **(B)** The efficiency of RNA interference by siRNA-1 and siRNA-2 on endogenous ARHI in PHPC was evaluated by western blot, compared with control. **(C)** ARHI-negative PHPC were treated with 10 μM DAC. DAC and siRNA downreglated ARHI were combined in rescue experiment. **(D)** The transfection was evaluated by western blot. **(E)** Cells were promoted by both siRNA-1 and siRNA-2, not control. **(F)** The expression of ARHI was then evaluated by RT-PCR. Untreated PHPC were employed as control (0 hr). The cell viability measured by CCK-8. All experiments were performed at least thrice and these points represent the average values, where error bars (mean±SD) are included, * indicates *P*<0.05, ** indicates *P*<0.01, two-sided paired *t* test.

We then wondered whether ARHI exhibits biological characteristics of a TSG in PCC *in vitro*. To evaluate the effect of ARHI on cell viability, ARHI-negative PHPC were transiently transfected with ARHI expression vectors, followed by a time-course assay. Significant proliferation inhibition was observed when compared with control (Figure 3A and 3D). To assess further the effect of loss of ARHI *in vitro*, ARHI-positive PHPC were transfected with ARHI-siRNA to knockdown ARHI, and the efficiency of the siRNAs was determined by western blot (Figure 3E). The PHPC treated with ARHI-siRNA-1/2 exhibited a significant increase in the cell proliferation rate when compared with the control siRNA or mock-treated cells (Figure 3B). The RT-PCR showed that the application of DAC induced the expression of ARHI mRNA and inhibited cell proliferation (Figure 3C and 3F). Furthermore, the latter effect was reversed with the introduction of ARHI-targeted siRNA in combination with DAC treatment (Figure 3G and 3H). Taken together, these data indicate that ARHI exhibited a specific inhibitory effect on PHPC proliferation *in vitro*.

### ARHI arrests the cell cycle and promotes PHPC apoptosis *in vitro*

**Figure 4:**
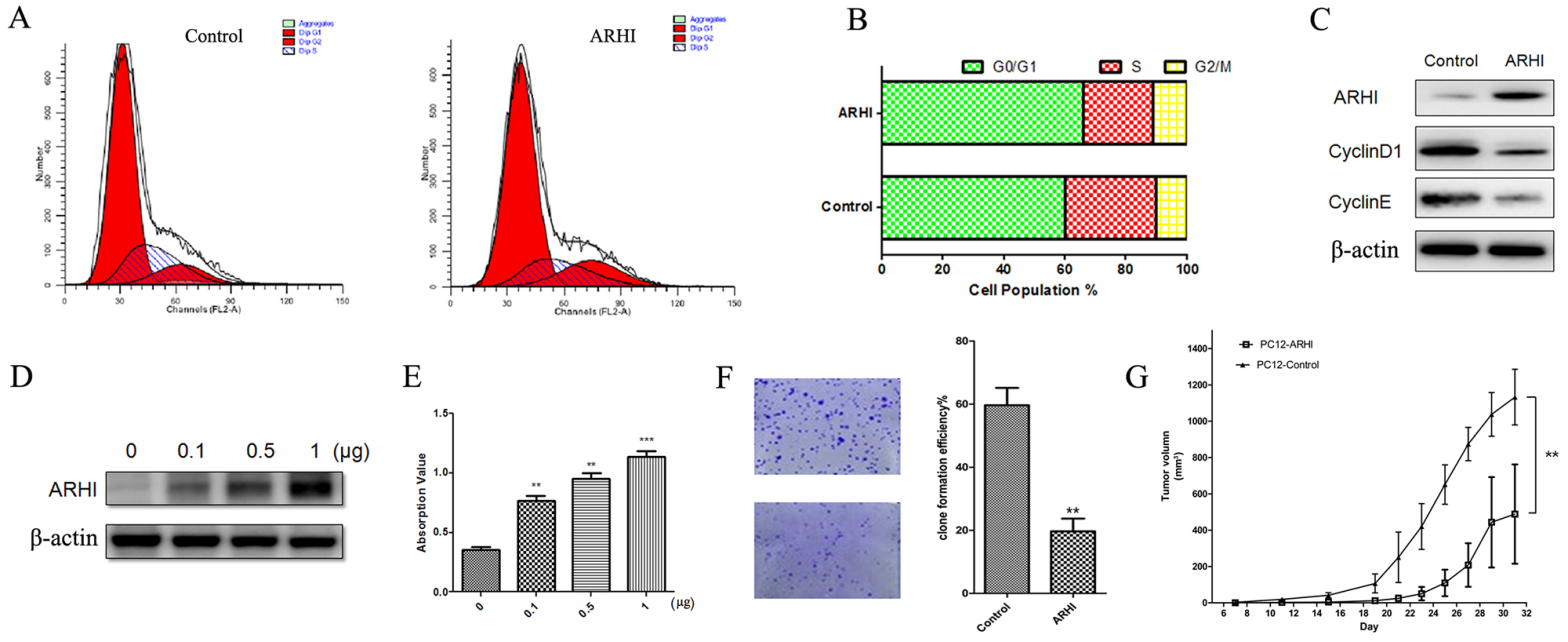
ARHI arrested cell cycle and promoted PHCP apoptosis **(A)** ARHI-negative PHPC were transfected with pcDNA3.1-ARHI; after 48 h, the PHPC were analyzed by FCM, and the G0/G1, S and G2/M phases of the cell cycle were tested. **(B)** Schematic analysis of cell population in different phase in Figure 4A. **(C)** CyclinD1 and CyclinE were also detected by western blot. **(D)** and **(E)** ARHI-negative PHPC were transfected with pcDNA3.1-ARHI. Apoptosis was determined by a Cell Death Detection Kit; ARHI expression promotes PHPC apoptosis in a dose-dependent way. ARHI expression was detected by western blot. **(F)** Images and the colony formation rates of control cell and ARHI overexprssed PC12 colonies after culture in soft agarose 14days. **(G)** Anticancer activity of ARHI *in vivo*. Nude mice were injected with 7×107 PC12 or PC12 stable cell clone with ARHI overexpression. Visible tumor size were measured and plotted. The error bars are represented as mean ± SD, * indicates P<0.05, ** indicates *P*<0.01, *** indicates *P*<0.001, two-sided paired *t* test.

In the above experiments, we showed obvious inhibition of PHPC proliferation by ARHI, and we further investigated the effect of its overexpression on the induction of cell cycle progression. Flow cytometry analysis showed that the percentage of S phase cells decreased (from 22.95% to 17.40%) whereas the G0/G1 phase cells increased (from 59.98% to 65.92%) and G2/M phase cells were almost unchanged (from 10.28% to 11.12%) after transfection of PHPC with ARHI for 48h. This indicates that the cell cycle was arrested at the G0/G1 phase by ARHI (Figure 4A and 4B). To confirm the cell cycle analysis by flow cytometry, important cell cycle checkpoint markers CyclinD1 and CyclinE were detected. The results showed that CyclinD1 and CyclinE were obviously decreased in cells showing ARHI overexpression (Figure 4C). These data show that ARHI arrested the cell cycle at G1/S. To further validate the role of ARHI in PCC, ARHI overespression plasmid was used to construct stable transfection in PC12cells, and then these cells were tested for colony formation. Consistent with our results from PHPC, we found that ARHI overexpression decreased the clone formation compared to the control group (Figure 4F). Xenograft experiment *in vivo* also confirms that ARHI worker as a tumor suppressor in PCC tumorigenesis (Figure 4G).

Given that failure of developmental apoptosis has been believed to be an important mechanism in PCC pathogenesis [33], the downregulation of ARHI in PCC may be hypothesized to allow cells to escape apoptosis. Therefore, we determined the effect of ARHI on apoptosis in PHPC. To quantify apoptotic cell death, a Cell Death Detection ELISA for fragmented DNA was used. The results showed that, when cells were measured at 48h after transfection with pcDNA3.1-ARHI in ARHI-negative PHPC or control vector, the two groups exhibited different levels of cell apoptosis in a dose-dependent way (Figure 4D and 4E). Unexpectedly, we did not observe apoptosis inhibition in PHPC with endogenous ARHI downregulated by siRNA (data not shown).

### ARHI downregulates p-ErK1/2 and p-AKT in PHPC

**Figure 5:**
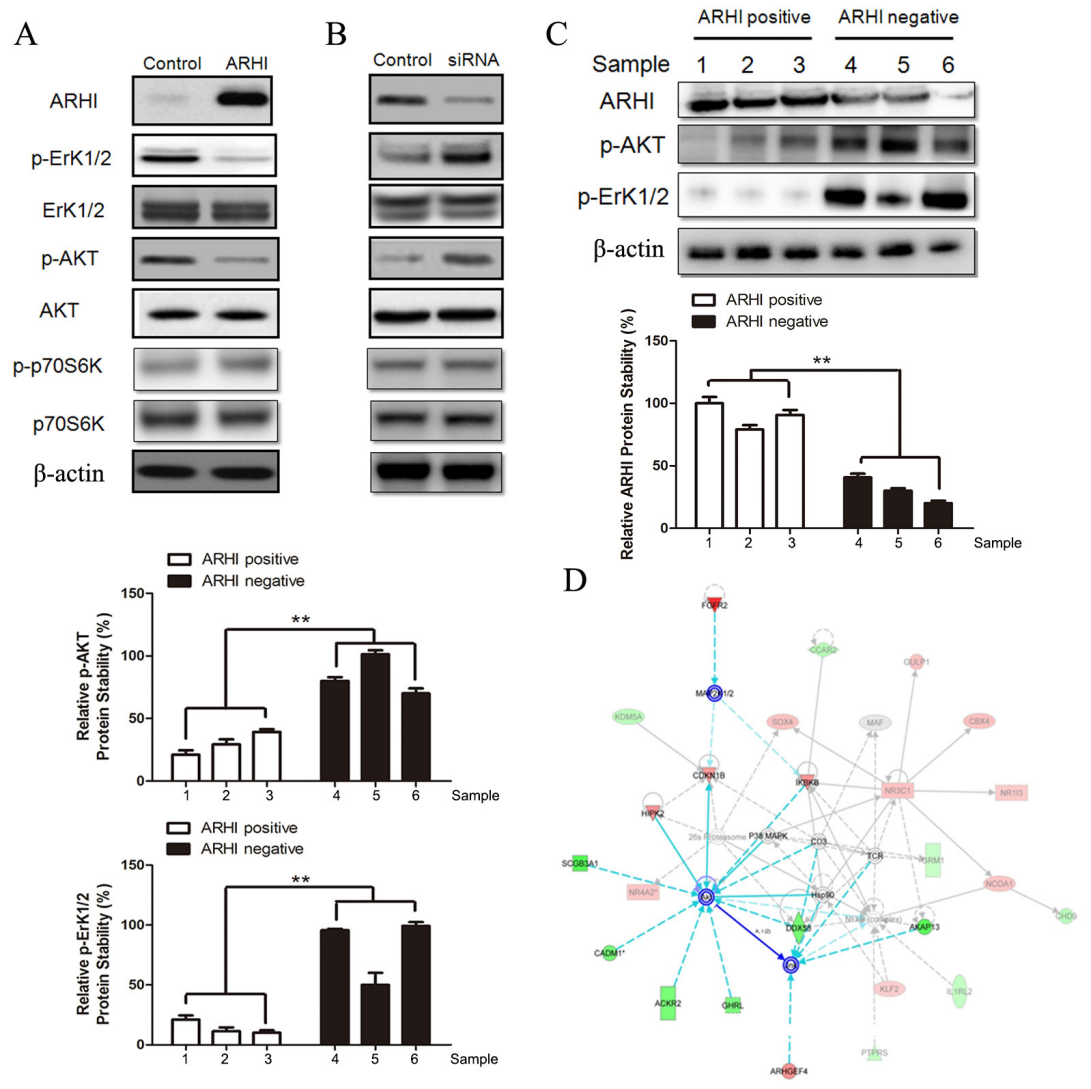
ARHI re-expression inhibits the PI3K/AKT and Ras/ERK signaling pathways ARHI re-expression in **(A)** negative-ARHI and **(B)** positive-ARHI cells decreases basal levels of p-AKT and p-ErK1/2. PHPC were transfected with pcDNA3.1-ARHI or siRNA for 48 h to overexpress or knockdown ARHI expression. Cell lysates were prepared and detected with the indicated antibodies by western blot analysis. Band intensities were quantified and compared with that of the respective control. **(C)** Each three ARHI-positive and -negative PCC samples were lysed to detect p-ErK1/2 and p-AKT by western blot. **(D)** Up and downregulated genes in PHPC with exogenous ARHI, compared to ARHI-negative PHPC, were imported into IPA. Through the core analysis, the top 10 associated networks were found, the first and the only meaningful pathway is shown in the figure. Red shows the upregulation genes and green presents the downregulated genes in the microarray. The obvious line links the AKT, CDKN1B and Junk pathways.

Unsupervised cluster analysis of genome-wide expression performed on hereditary PCC has revealed two dominant groups associated with kinase receptor signals, which have been linked to NF1 and RET mutants (the first cluster) and hypoxia and angiogenesis through stabilization of hypoxia-inducible factor alpha (the second cluster), which occurs in all VHL- and SDHx-mutant tumors [12, 30, 34]. Recent result showed that sporadic PCC always belong to the first cluster [32]. We detected the key pathways associated with the first cluster, which include RAS-ErK1/2, PI3K-AKT, and mTOR. In these pathways, p-ErK1/2 and p-AKT were decreased in PHPC with negative ARHI expression after ARHI transfection, but p-p70S6K was not changed (Figure 5A). Knockdown of ARHI in PHPC with endogenous ARHI confirmed the above result (Figure 5B). Furthermore, protein was extracted from three fresh PCC samples with normal or deleted ARHI copy number to detect p-ErK1/2 and p-AKT. The western blot data showed that p-ErK1/2 and p-AKT were increased significantly in ARHI-negative PCC samples (Figure 5C). In addition, to understand the global transcriptional disturbance, we used expression microarray analysis by IPA. It was found that molecules with fold change>|1.5| were most enriched in PI3K-AKT, CDKN1B (p27^Kip1^) and Junk pathways (Figure 5D).

### ARHI increased p27^Kip1^ mRNA and protein stability

**Figure 6:**
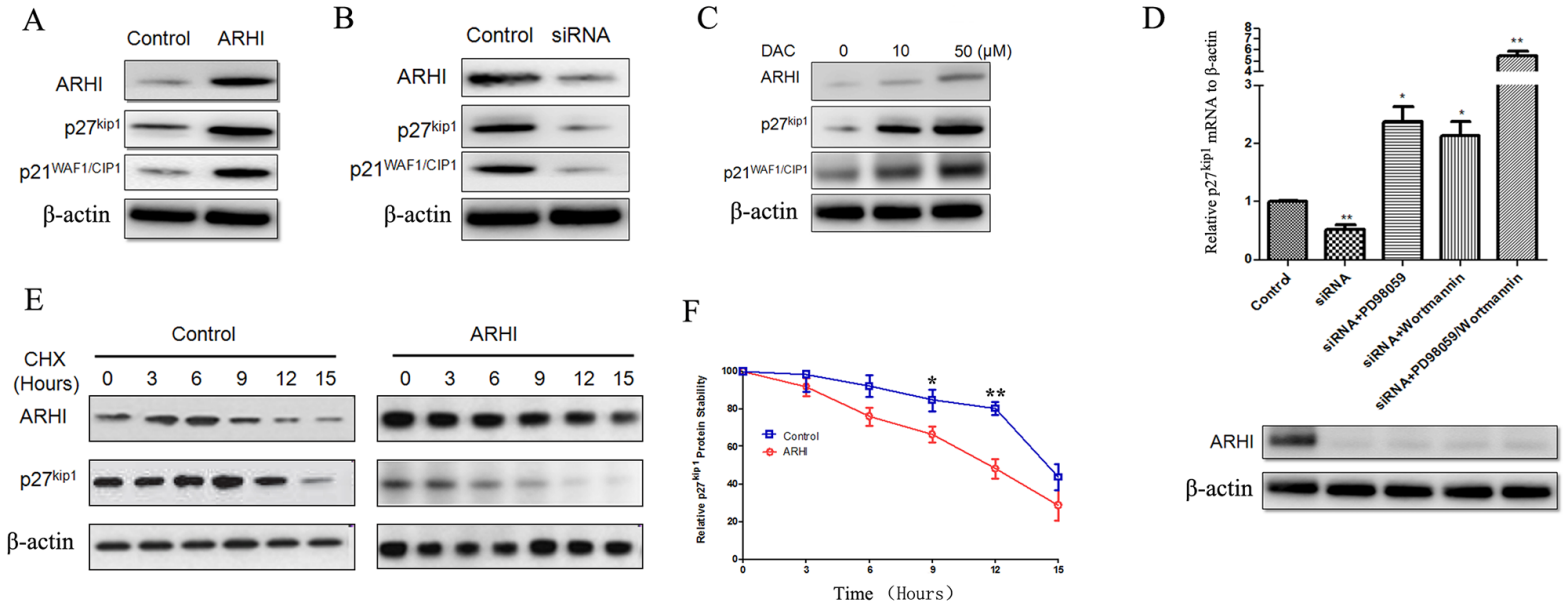
ARHI increased p27Kip1 mRNA and protein stability **(A)** and **(B)** ARHI-negative PHPC caused to overexpress ARHI, and ARHI-negative PHPC with knockdown of ARHI; 48 h after transfection, p21^WAF1/CIP1^ and p27^kip1^ were detected by western blot. **(C)** ARHI-negative PHPC were treated with DAC in different concentrations; p21^WAF1/CIP1^ and p27^kip1^ were detected by western blot. **(D)** ARHI-positive PHPC with ARHI knockdown with siRNA, treated with Ras-pErK1/2 and PI3K-AKT pathway-specific inhibitors for 24 h. The p21^WAF1/CIP1^ and p27^kip1^ mRNA and RT-PCR. ARHI expression in different treatment groups was detected by western blot. **(E)** ARHI expression correlates with decreased protein stability of p27^Kip1^. PHPC negative for ARHI were transfected with pcDNA3.1-ARHI for 48 h and subsequently treated with cycloheximide (CHX) at a final concentration of 50 μg/ml and samples were harvested at 0, 3, 6, 9, 12 and 15 h time points after the addition of CHX. Band intensities were measured by Image J. Normalization was done by dividing the p27^Kip1^ signal by the ACTB signal. *P* values were measured by Student’s t test, * indicates *P*<0.05, ** indicates *P*<0.01. The results are presented as mean ± SD. **(F)** Quantization of p27^Kip1^ protein levels to determine relative stability in the absence or presence of ARHI expression. Results for both panels are from a minimum of three independent experiments.

Having evaluated the data from our cell cycle arrest, microarray and pathway analyses, we investigated whether ARHI regulated cell cycle inhibitors such as p21^WAF1/CIP1^ and p27^Kip1^in PHPC. ARHI overexpression promoted the expression of p21^WAF1/CIP1^ and p27^Kip1^ simultaneously (Figure 6A), and ARHI knockdown confirmed this result (Figure 6B). We also treated PHPC with negative ARHI with DAC, to upregulate endogenous ARHI, and p21^WAF1/CIP1^ and p27^Kip1^ expression were also increased (Figure 6C). We already know that re-expression of ARHI in cancer cells inhibits signaling through the Ras/MAP pathway, induces p21^WAF1/CIP1^, and downregulates cyclin D1. Therefore, we also sought to determine how ARHI increased p27^kip1^. First we examined the p27Kip1 mRNA level, and found that ARHI regulated p27^Kip1^ mRNA transcription through both the Ras/pErK and PI3K-Akt pathways (Figure 6D). In older to address the possibility that ARHI may also increase p27^Kip1^ protein stability, ARHI-negative PHPC were induced to overexpress pcDNA3.1-ARHI for 48 h. The remaining cells were treated with cycloheximide (CHX), and harvested at various time points. ARHI expression was sufficiently induced after 48 h, and was maintained thereafter (Figure 6E). Irrespective of treatment, there was no difference in p27^Kip1^ levels at the 0h time point. A gradual decrease in p27^Kip1^ expression was observed in control cells, with a p27^Kip1^ half-life of approximately 12h, and ARHI prolonged p27^Kip1^ degradation at 9–12h after CHX treatment (Figure 6F).

## DISCUSSION

Previous studies by different research groups have shown that several types of DNA genomic variation, especially deletions, are frequently found in PCC [17, 24]. Of these deleted regions, 1p36, 1p32–31, 1p22–21 and 1p13 were considered the most common sites of loss in PCC [17, 18]. Proximal to 1p36 there are three TSGs, namely SDHB, KIF1βand PRDM2, associated with PCC [9, 35, 36]. However, the tumor suppressor in other regions has not been found to date. ARHI, a maternally imprinted gene located on 1p31.3, is expressed mono-alleically from the paternal allele in normal cells. Previous studies have shown that ARHI expression is downregulated in many cancers, including breast carcinomas, ovarian cancers, and others [20, 23, 29, 37-40]. ARHI is known to be an imprinted tumor suppressor gene. Loss of ARHI expression can occur through genetic events such as the loss of one allele, and/or through epigenetic events, such as alteration of the patterns of DNA methylation, histone deacetylation, and histone methylation, in breast cancers and ovarian cancers [27, 28, 41, 42]. However, the functions and mechanisms of the ARHI gene in PCC are unclear. Loss of chromosome 1p is an important genetic event in sporadic PCC tumorigenesis [17-19, 24, 26]. Previous research has shown that the functional allele of ARHI is lost in 40% of ovarian and breast cancers, and in 69% of follicular thyroid carcinoma [23]. In this study, we used qRT-PCR and FISH, which investigated copy number, to evaluate the allelic imbalance of the ARHI locus, and 40 of 44 sporadic PCC had this signature. These data imply that copy number deletion is a very common genetic aberration at the ARHI locus in sporadic PCC. Furthermore, we found that ARHI was downregulated significantly in nearly all PCC cancerous tissues, when compared with non-PCC tissues (Figure 1A and 1E). In addition, we also confirmed that ARHI has usually lost a functional allele and may even be negatively expressed in sporadic PCC (Figure 2B).

DNA methylation and histone modifications are most important epigenetic hallmarks of human cancer [43]. However, possible epigenetic inactivation of the sporadic PCC predisposing genes by promoter methylation has so far not been fully determined [44, 45]. Previous research has shown that the promoter CpG islands of NF1, RET, SDHB, VHL, SDHD, SDHAF2, TMEM127and MAX were unmethylated in PCC [13, 45-47]. But recent research uncovered new epigenetic events in PCC, the results showed that VHL epigenetically inactivated in PCC [48]. This finding suggests that TSG epigenetic inactivation in sporadic PCC tumorgenesis might be further indentified [48]. Drugs targeting epigenetic pathways might constitute useful alternative treatments in PCC [44, 49]. ARHI as an imprinted TSG in breast and ovarian cancer, unlike the known susceptibility TSGs in PCC, mutation is likely not the primary mechanism of ARHI inactivation. Indeed, we observed concurrent hypermethylation of promoters, including all three CpG islands, and genome copy number deletion in sporadic PCC. In our study, ARHI promoter hypermethylation was observed in 89.4 % (34/38), copy-number deletion in 90.9% (40/44) and underexpression in almost all of the PCC samples. There was a negative correlation between ARHI promoter methylation ratio and its mRNA expression. Moreover, through the DAC experiment, we found that ARHI expression could be recovered in ARHI negative PHPC, although the mechanism of ARHI methylation is yet to be unravelled. These results show that the methylation of ARHI CpG islands regulates its expression in PHPC. It is unknown, however, whether the hypermethylation of ARHI has any pathological consequences in PCC, our data demonstrated that overexpression of ARHI inhibits cell growth in ARHI-negative PHPC, while silencing expression of the endogenous ARHI gene can promote cell proliferation in ARHI-positive PHPC. The results suggest that the downregulation of ARHI may contribute to tumorigenesis via inhibition of cell growth, which is triggered mainly by epigenetic events in PCC specimens.

12 susceptibility genes in PCC belong to two clusters. One displays activated hypoxia and pseudohypoxic signals even in normoxic conditions, while the other upregulates the RAS/RAF/MAPK, PI3/AKT and m TOR signaling pathway [8, 30, 31, 33, 34, 50, 51]. Susceptibility gene dysfunction, for example somatic mutation, could explain why this sample of PCC tumors clustered into the two expression sets. Another important possibility is the existence of novel TSGs that shared a mechanism with susceptibility genes in PCC tumorigenesis. Most sporadic PCC without germline mutations in known susceptibility genes are found in the latter cluster [32]. In our research, ARHI inactivation led to the sustained activation of Ras/MAP and PI3K/Akt signaling pathways, which are the most important pathways controlling adrenal medullar cell proliferation. We used microarray data to uncover the signal pathways disturbed by ARHI in PHPC. The results showed that the MAPK, pAKT, p27^Kip1^ and Junk pathways were altered in the group showing ARHI overexpression. Overexpression of ARHI in PHPC may be associated with decreased expression of p-AKT, pErK1/2 and increased p27^Kip1^; all these effects will decrease cell proliferation. The next experiment suggested that ARHI could inhibit the proliferation of PHPC, leading to cell cycle arrest in the G0/G1 phase, which is possibly due to a reduction in cell cycle inhibitors downstream of the ARHI signal. Overexpression of ARHI in cancer cells inhibits signaling through the Ras/MAP and PI3K-AKT pathways, induces p21^WAF1/CIP1^, and downregulates cyclin D1 [21]. We also showed for the first time that ARHI regulates p27^Kip1^ stability. The research using disease models shows that 95% of p27^Kip1^ knockout mice develop PCC [52], and nearly half of human PCC samples were found to have decreased p27^Kip1^ [53]. Therefore, it is very important to find out how ARHI regulates the negative cell cycle regulatory factor p27^Kip1^ in PCC tumorigenesis. Expression of p27^Kip1^ mRNA was suppressed in PHPC overexpressing ARHI by the Ras/MAP and PI3K-AKT pathways, and p27^Kip1^ protein stability was significantly elevated in such PHPC. These changes may result in cell cycle blockage of PHPC overexpressing ARHI; however, the specific mechanisms remain to be further explored. Based on our studies, we propose that human chromaffin cells escaped from the normal surviving progress that is supervised by ARHI to be potential to proliferate.

As we known, indeed, the research indicated that failure of developmental apoptosis was believed to be an important mechanism in PCC pathogenesis [33], so we suspect that ARHI downrgulation may contribute to escape apoptosis, in order to validate this hypothesis, we identified some important cellular signaling pathways and cellular behaviors such as apoptosis and cell cycle progression (Figure 5D), but we only show the proliferation inhibition not apoptosis alteration in the siRNA treatment group after multiple repeatable detections. Possible reasons including: 1. Both proliferation and apoptosis are the cause of PCC tumors, in considerable detections, it is natural not to observe these results in the same treatment simultaneously. 2. Tumor’s progress contains a very complicated molecular network, it is easy to understand that only one molecule downregluation may not be sufficient to alter the apoptosis signal pathway. According to our microarray result, we suspect that proliferation inhibition is probable a major mechanism in PCC pathogenesis regulated by ARHI, and maybe some molecules compensate the apoptosis effect of ARHI depression in PCC. 3. Escape apoptosis regulated by ARHI maybe needed an exogenous activation to amplify the detection signal in PHCP, we tired our best to increase the PHPC apoptosis, but as the primary cell from the PCC patient, PHCP is very sensitive to the exogenous activation, even it is under the extremely low concentration of exogenous activation, PHCP is almost died. Under these circumstances, we could not indicate accurate relationship between ARHI and PCC tumor apoptosis. So we will continue to optimize PHCP culture to clarify other the functions of ARHI in PCC tumorigenesis in further.

In summary, our study showed that ARHI is involved in PCC tumorigenesis as a novel tumor suppressor at several different levels, including (1) loss of a functional allele in PCC, (2) hypermethylation of promoter CpG islands in PCC, (3) cell cycle arrest and promotion of PHPC apoptosis, (4) inhibition of proliferation through a decrease in Ras-ERK/AKT signaling, (5) enhanced expression and increased protein stability of p27^Kip1.^ The results will provide opportunities for the development of robust assays for ARHI as a clinical diagnostic index or as a potential target for epigenetic therapy in sporadic PCC.

## MATERIALS AND METHODS

### Patients and samples

The study included a total of 44 tumors from patients with sporadic adrenal PCC, who were operated on between 2012 and 2014 in Peking Union Medical College Hospital. The clinical data for the patients are summarized (Table 1), and their detailed characteristics are listed (Supplementary Table 1). Fresh PCC samples were obtained from patients and immediately immersed in RNAlatter before being transferred to –20°C for storage. All the research was conducted in accordance with ethical guidelines.

**Table 1:** Summary of PCC sample

Variable	Number or mean±SD
Age at diagnosis (year)	44 ±15
Sex (F/M)	20/24
Benign/Malignant	44/0
Sporadic/Truly sporadic	44/34
Adrenal/Extra-adrenal	40/4
Tumor diameter (cm)	4.4±1.6

### Microarrays and ingenuity pathways analysis

The gene-expression profiling data were analyzed using RNA from overexpressed ARHI of PHPC and control cells with negative ARHI expression on Affymetrix HG-U133 plus 2.0 microarrays. Details regarding sample preparation, hybridization, and image acquisition have been described in the manufacturer’s instructions. The raw data were processed using the MAS5 algorithm, and probe sets that did not show expression (called “absent” by MAS 5.0) in any sample were removed. The CEL files were imported into Partek Genomics Suite 6.0, and data were normalized using the RMA algorithm. A one-way ANOVA was used to analyze the changes in expression by comparing the PCC and respective matched control. The genes significantly underexpressed in all four paired samples were selected for further research.

The expression profile data obtained from one-way ANOVA were further analyzed. Genes were selected that were either induced (fold change >1.5; called “present”) in all normal tissues or repressed (fold change <-1.5; called “present”) in all PCCs. This gene set was imported into Ingenuity Pathways Analysis (IPA). Gene networks representing key genes were identified using the curate IPA Knowledgebase.

### Quantitative RT-PCR

RNA was extracted from the cell lines using Trizol (Invitrogen), following the manufacturer’s recommended protocol. Total RNA (1 μg) from each sample was reverse transcribed using oligo d(T) primers and Superscript III reverse-transcriptase (Invitrogen). Gene-specific primers were designed with Primer Premier 5.0 Software (Premier, Canada) and qPCR was performed using FastStart SYBR Green Mix (Takara) on the ABI step plus one (Life Technology) to determine the relative levels of expression of ARHI and β-actin in all samples. The reactions were run in triplicate in the ABI step plus one.

qRT-PCR was also used to confirm copy number, using primers that amplify across an exon for ARHI and control genes. The NCBI PRMER-BLAST online tool (http://www.ncbi.nlm.nih.gov/tools/primer-blast/index.cgi?LINK_LOC=BlastHome) was used to decrease the probability of cross hybridization of primers. qRT-PCR was carried out with 10 ng per reaction of the same genomic DNA as that used in the promoter analysis. All samples were then normalized to C2 and the copy number in the blood samples was set to 2. A copy number relative ratio <1.4 was represented copy number deletion, and a copy number relative ratio >2.6 was recorded as amplification.

### Mutation analysis

We detected germline mutations of RET, VHL, SDHx, FH, MAX and TMEM127 with direct Sanger sequencing, as described previously [54-56] or with newly designed primers (Supplementary Table 2). For each reaction, 30 ng of tumor DNA was PCR-amplified for 35 cycles using HotStar Taq Polymerase (Takara). Sequences were analyzed by alignment to the UCSC sequence using the DNAMAN6.0 tool (LynnonBiosoft). Somatic mutations in NF1, HIF2A, RET, VHL and SDHB were detected simultaneously using next generation sequencing (BGI, Beijing).

### Sodium bisulfite modification, EpiTYPER methylation analysis

Genomic DNA was bisulfite-treated using an EZ DNA Methylation Kit™ (Zymo Research). Quantitative DNA methylation levels were determined as previously described using the MassARRAY EpiTYPER system (SEQUENOM Inc.), a mass spectrometry-based method. The average methylation of their CpG islands was respectively calculated to give the ARHI methylation levels for all control and patient samples. Specific primers were designed using “SEQUENOM Online Tools” (http://www.epidesigner.com/start3.html) as previously described.

### Cell culture, transfection

Pure PCC tumors with no mutations in susceptible genes were cut into pieces. After washing 8-10 times with pre-cooled Hanks balanced salt solution (HBSS), the rinsed pieces were put into the dissociation solution (2.6 g/ml collagenase I solution, 0.15 g/ml Hyaluronidase Type I-S and 0.01% DNase I). Samples were incubated in a water bath for 35- 45min at 37°C with stirring at 300 rpm. The digestion was stopped with 1% heat inactivated fetal bovine serum as soon as the solution turned cloudy. The solution was mixed gently with a Pasteur pipette and filtered with a 300 mesh (49 μm) sieve. Dissociated cells were centrifuged at 700 rpm for 3-5 min to form a pellet and then resuspended in a culture medium comprised of DMEM supplemented with 15% FBS serum. The PHPC were plated in 6 well plates coated with poly-D-lysine at 37°C in 5% CO2. Two hours later, the dishes were gently flooded and then refilled with culture medium. After 24 h, the medium was resuspended very gently using a pipette in order to collect the suspended cells, which were mostly PHPC. The fibroblasts that were closely adherent to the bottom of the wells, were discarded. ARHI-negative and -positive PHPC were collected from every set of three mixed fresh samples of sporadic PCC, which confirmed that the ARHI copy number was normal or deleted.

Cells were transfected with control or targeted siRNAs using the X-tremeGENE HP DNA Transfection Reagent (Roche). Transfection of HPHC was performed following the standard manufacturer’s protocol. Briefly, siRNA (100 nM final concentration, ON-TARGETplus DIRAS3 siRNA, L-008660-00-0005, Dharmacon) and transfection reagents were incubated for 20 min at room temperature. The transfection reagent was added to cells and allowed to incubate for 48 h before the cells were harvested and analyzed for protein and RNA expression.

### Treatment with DAC and bisulphite sequencing

PHPC were grown for 0–96 h before treatment with 10 μM 5-aza-2′-deoxycytidine (DAC) (Sigma) dissolved in dimethylsulfoxide. The DNA was extracted and analyzed by bisulfite treatment, as described above. The gDNA was isolated from identically treated cultures for quantitative RT–PCR analysis. The control samples were treated with the dimethyl sulfoxide vehicle only. For bisulfate sequencing PCR reactions, specific primers were designed using “Methprimer” (http://www.urogene.org/methprimer/index1.html) or were used as previously described. The amplified fragments were cloned using pGEM-T Easy (Promega). After cloning, 12 clones from each sample were randomly selected for DNA sequencing. Inserts were sequenced using T7 primers.

### Analysis of survival, cell cycle and apoptosis

Cell viability was determined using a Cell Counting Kit-8 (CCK8) (Dojindo). For the cell cycle assay, cells were subjected to DNA staining by propidium iodide. The percentage of cells for each different cycle phase was determined by FACS analysis. Analysis of apoptosis was done using a Cell Death Detection Kit (Roche) and a cell death detection ELISA, according to the manufacturer’s instructions. All experiments were repeated three times and average values are reported. Cell colony formation assays were performed using 6-plate-well coated with 0.5 ml bottom soft agar mixture. PC12 cells overexpressed with ARHI or not were separately mixed with top agar (DMEM, 20% FBS, 0.3% soft agar) and seeded into each plate, after the bottom layer had solidified. Two weeks later, the colonies were fixed with methanol and stained with 0.5% crystal violet. The number of colonies (>50 cells) was counted on an inverted microscope.

### Xenograft experiment *in vivo*

20 BALB/c nude mice 4–6 weeks old were prepared for animal xenograft experiment. Then 0.2 ml PC12 (with ARHI overexpression or not) suspended in PBS at a density of 7×10^7^ were injected into each mice subcutaneously in the flank back skin. One group was injected PC12 with overexpressed ARHI, the other group was injected with normal PC12. Tumor volumes were routinely measured every 3 days by width and length using caliper. Tumor volume = (length×width^2^)/2. After 31 days, the xenograft tumors were taken out and weighed quickly then the data were recorded for statistical analysis.

### Protein extraction and immunoblotting

For western blot analysis, lysates were size fractionated by SDS-PAGE and transferred onto PVDF membranes. Antibodies recognizing ARHI were obtained from Lifespan (LS-C167421). All the other antibodies were from Cell Signaling (CST). After secondary antibody incubations, signals were detected by enhanced chemiluminescence. For the IHC assay, surgically resected PCC tissues were collected from 2012 to 2014 with approval by the Peking Union Medical College Hospital. To calculate the level of the positive signal, four fields of one sample were assessed and the results were expressed as the percentage positive area. All samples were analyzed at the same threshold values.

### Fluorescence in situ hybridization

Cytospin slides were fixed in methanol and acetic acid (3:1 ratio) for 15 min, dehydrated in a graded (70–100%) series of ethanol, and air dried. The fluorescence labeled probes were used for hybridization to band 1p31.3 at the ARHI genomic locus (STSG-34496: SHGC-57898, red) and to band 1q21 (SHGC-145644: SHGC-147087, green) of human chromosome 1, according to the manufacturer’s instructions.

## SUPPLEMENTARY MATERIALS FIGURES AND TABLES






