# Supplementary material for: ARHI is a novel epigenetic silenced tumor suppressor in sporadic pheochromocytoma
Source: Oncotarget. 2017 Sep 21;8(49):86325–38. doi: 10.18632/oncotarget.21149 (PMC5689688; doi:10.18632/oncotarget.21149)
Supplement: Supplementary file 1 [file oncotarget-08-86325-s001.pdf]

ARHI is a novel epigenetic silenced tumor suppressor in sporadic pheochromocytoma

SUPPLEMENTARY MATERIALS

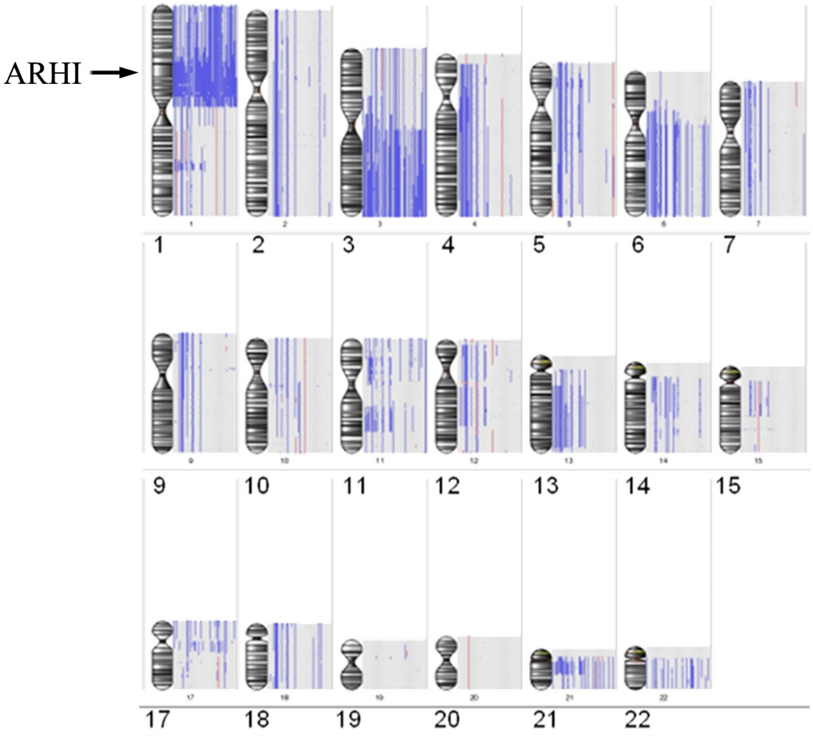

Supplementary Figure 1: BAC array comparative genomic hybridization (CGH) on 78 PCC tumors. Data form GSE38525 was re-analyzed by Partek 6.0. Each autosomal chromosome copy number variation (CNV) frequency is shown as a histogram; chromosomes X and Y are not shown. Red indicates copy number amplification; blue indicate copy number deletion. The height of the histogram is proportional to the number of samples showing the CNV in each chromosomal region. An arrow indicates the ARHI locus.

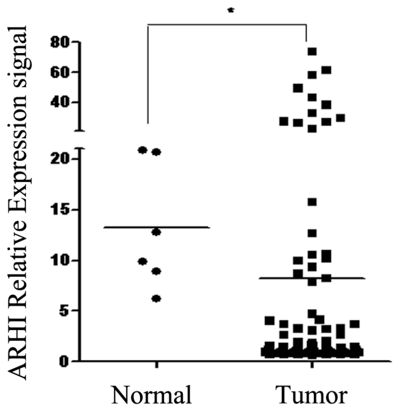

Supplementary Figure 2: ARHI mRNA expression signal in normal human adrenal tissues (n=6) and primary PCC (n=84). Normalized expression signal from E-MTAB-591 was re-analyzed by Partek 6.0. When compared with normal tissue, ARHI is significantly reduced in the PCC (p<0.0001, two-sided Mann Whitney test).

Supplementary Table 1: Clinical characteristic of the PCC/PGL patients.

See Supplementary File 1

Supplementary Table 2: PCR primers sequence.

See Supplementary File 2
